# Interlaminar and varicose-projection astrocytes: toward a new understanding of the primate brain

**DOI:** 10.3389/fncel.2024.1477753

**Published:** 2024-11-25

**Authors:** Caterina Ciani, Carmen Falcone

**Affiliations:** Department of Neuroscience, International School for Advanced Studies (SISSA), Trieste, Italy

**Keywords:** astrocytes, evolution, cerebral cortex, primates, interlaminar astrocytes, varicose-projection astrocytes

## Abstract

In the last years, science started to move toward a more *glio-neurocentric* view, in which astrocytes are hypothesized to be directly involved in cognitive functions. Indeed, astrocytes show a variety of shapes with species-specific characteristics, suggesting a specialization of roles during evolution. Interlaminar (ILA) and varicose-projection (VP-As) astrocytes show an anatomical organization that is different compared to the classical horizontal net typically formed by protoplasmic and fibrous astrocytes. ILAs show a modular architecture with the soma in the first cortical layer and processes toward the deep layers with species-specific length. VP-As reside in the deep layers of the cortex, are characterized by varicosities on the longest processes, and are individual-specific. These characteristics suggest roles that are more complex than what was theorized until now. Here, we recapitulate what we know so far from literature from the first time ILAs were described to the most recent discoveries, spanning from morphology description, hypothesis on the development to their features in diseases. For a complete glance on this topic, we included a final paragraph on which techniques and models were used to study ILAs and VP-As, and what new avenues may be opened thanks to more novel methods.

## Highlights

• ILAs and VP-As have primate-specific features.
• ILAs and VP-As have been found to be involved in neurological disorders.
• Investigating the functions of ILAs and VP-As can help understand primate brain complexity.

## Introduction

The brain’s complex abilities are the outcome of a synergic effort made by all the components of active units. Not only neurons but also astrocytes, oligodendrocytes, microglia, extracellular space, and its matrix collaborate for an all-inclusive model (Semyanov and Verkhratsky, 2021, 2022).

Therefore it is time to shift toward an inclusive concept of an active milieu, recognizing glial cells, together with all the other components, as co-main actors of the brain, actively collaborating with neurons (Colombo and Reisin, 2004; Oberheim et al., 2006; Semyanov and Verkhratsky, 2021, 2022; Verkhratsky and Nedergaard, 2014). Evidence now shows that astrocytes are directly involved in synaptic connections and control, ranging from ionic balance to the genesis and behavior of synapses (Eroglu and Barres, 2010; Oberheim et al., 2006; Reisin and Colombo, 2002; Semyanov and Verkhratsky, 2022; Verkhratsky and Nedergaard, 2014, 2018), roles far beyond what was theorized in the past (Colombo et al., 1995; Khakh and Sofroniew, 2015).

The mammalian brain is fundamentally organized into columns, traditionally seen as clusters of neurons (*columnar organization*) (Hubel and Wiesel, 1962, 1968; Reisin and Colombo, 2002). However, this new view of an active milieu in which the glia is as much fundamental as neurons is supported by evidence of a glial columnar organization, challenging the traditional perception of astrocytes as part of a horizontal cytoarchitecture forming a *panglial syncytium* (Colombo et al., 1995; Colombo et al., 1997c; Hern’ et al., 2002; Oberheim et al., 2006). Which relegate astrocytes to merely “diffusing” local perturbations or information to distant loci (Colombo, 2001).

The concept of functional glial columns in the adult brain is revolutionary and was described as a “modular structure” by Jorge Colombo (Colombo and Reisin, 2004). The only notable exceptions until now, are the ontogenetic columns created by the radial glia (RG) during mammalian embryonic development (Colombo et al., 1999, 2004). Further supporting this hypothesis is the observation that neuronal columns, which are evolutionary advantageous for increasing brain size and economizing on wiring, need to be isolated in functional units. This is ideally achieved by an organized glial compartment, a role that the atypical morphology of the interlaminar astrocytes (ILAs) seems to fulfill (Colombo, 2001). ILAs are a particular subpopulation of astrocytes positive to the marker GFAP^+^ with the cell body in layer I, and long inter-laminar processes perpendicular to the pia, recapitulating the columnar organization of neurons (Verkhratsky and Butt, 2023). These astrocytes interact with blood vessels, perivascular astrocytes, neurons, and protoplasmic astrocytes, suggesting a significant role in linking vertical and horizontal information flow (Sosunov et al., 2014). This putative integrative function is likely a key factor in complex cognitive abilities typical of mammals.

Another type of astrocyte has been observed in humans and apes only: the varicose-projection astrocytes (VP-As) (Falcone et al., 2022). These astrocytes show peculiar varicosities along their longer processes and are present in the deeper layers of the cortex and white matter. Like ILAs, their function is still unknown. It has been hypothesized that they might constitute a primate-specific response to certain brain conditions (Falcone et al., 2022).

Together with neurons, both ILAs and VP-As might be crucial players in regulating primate brain complexity and might represent the key to better understand brain evolution. Here we will provide a comprehensive review of the findings regarding ILAs history, morphology, development, and role in health and disease together with the limited knowledge available on VP-As.

## History

Glial history starts in the 19th century when it was becoming evident that the neural tissue of the brain was sustained by a “nerve connective tissue” (Chvátal and Verkhratsky, 2018). More recent tissue staining techniques helped scientists evolve a new concept of syncytium in which glial cells are interconnected through gap junctions (Verkhratsky and Butt, 2023).

**FIGURE 1:**
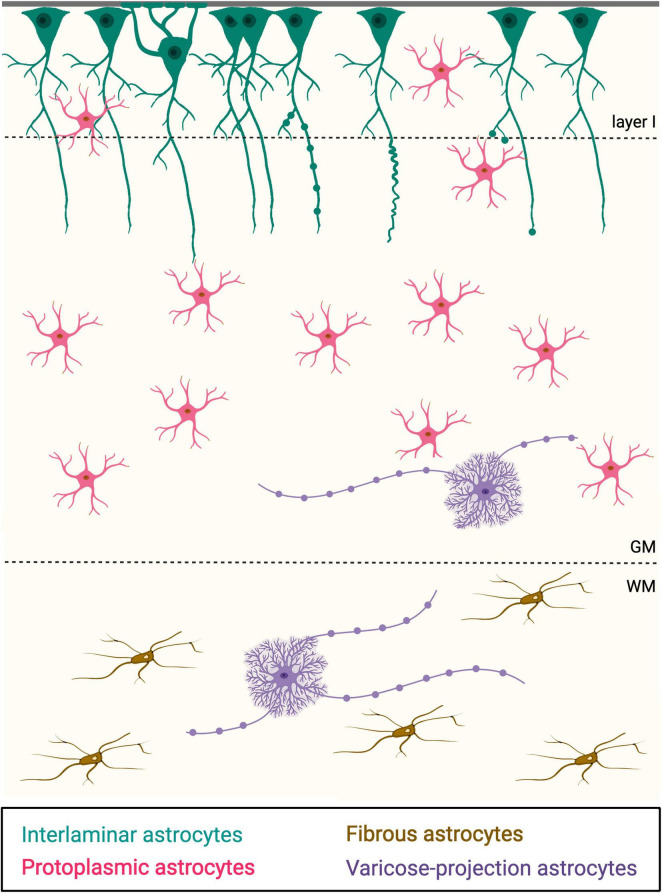
Schematic representation of the four main types of astrocytes recognizable based on their morphology. In teal, interlaminar astrocytes; in magenta: protoplasmic astrocytes; in brown, fibrous astrocytes; in violet, varicose-projection astrocytes. Pia is on top. GM, gray matter; WM, white matter.

From the late 19th century, our comprehension of glia’s abilities, such as maintaining the blood-brain barrier, homeostasis, and neurotrophic support of the neurons, improved significantly (Colombo and Reisin, 2004). During this period, canonical astrocytes were classified by Andreizen and Kolliker as protoplasmic (in the gray matter) or fibrous (in the white matter) (Figure 1), according to their location (Besser, 1866; Kolliker and Albert, 1889; Oberheim et al., 2009). The term “*astrocytes*” refers to the typical star-like morphology with short processes (less than 90 μm) that create connections with surrounding cells, resulting in a *single-cell spherical domain* that does not overlap with other astrocytes. This creates a well-connected network that controls the homeostasis and metabolism of the brain in a cooperative manner (Colombo et al., 1997a; Falcone and Martínez-Cerdeño, 2023; Oberheim et al., 2009).

**FIGURE 2:**
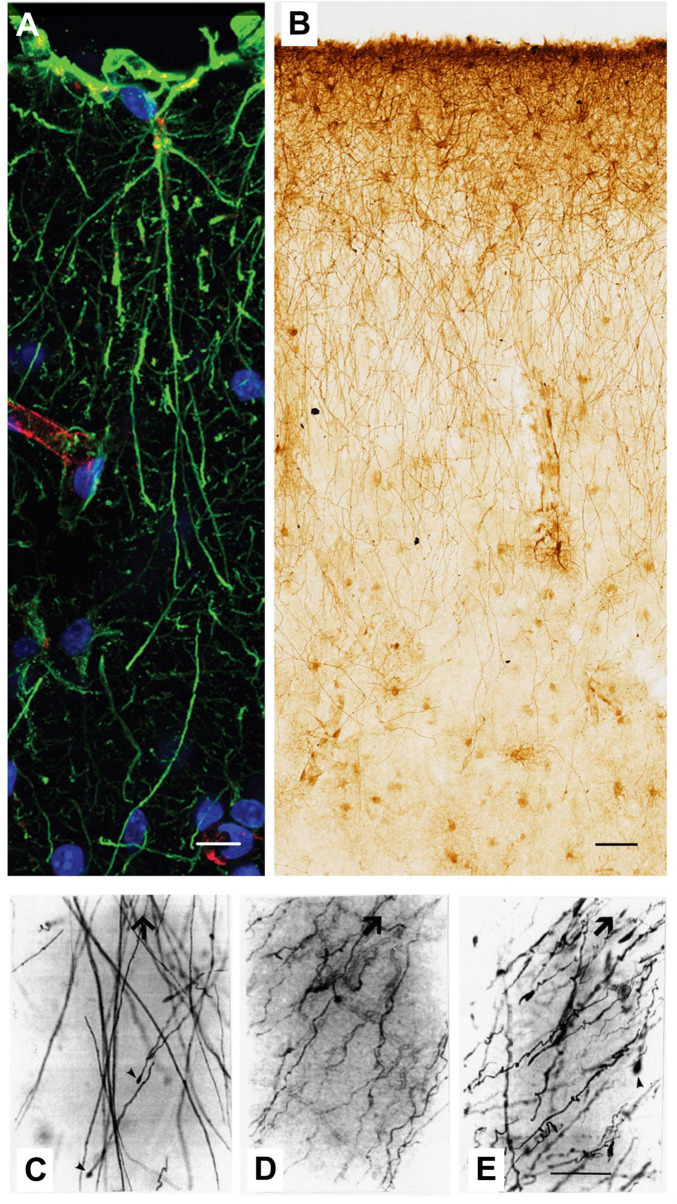
Example of GFAP+ interlaminar astrocytes and their processes from the cerebral cortex of primates [Adapted from Falcone et al. (2019) and Reisin and Colombo (2002)). **(A)** Example of an ILA in the prefrontal cortex of rhesus macaque (*Macaca mulatta*), with pia on top of the image. Immunofluorescence staining: DAPI in blue, GFAP in green, Lectin in red. Scale bar = 20 μm. **(B)** Representative image of GFAP+ pial ILAs in bonobo (*Pan paniscus*) dorsal cortex, immuno-stained with DAB. Scale bar = 50 μm. **(C–E)** Arrowheads indicate the cytoskeletal terminal masses decorating the endings of ILA processes, immunostained with anti-GFAP antibody. Large arrows indicate the direction of the cortical surface. **(C)** Brodmann Area 10, age not available; **(D)** Brodmann Area 17/ 18, age 65; **(E)** Brodmann Area 4, age 44. Scale bar in **(C)** = 18 um; scale bar in **(D,E)** = 20 μm.

Astrocytic networks work synergically, thanks to the *gap junctions* that connect astrocytes, opening or closing according to the extracellular factors (Colombo, 2001; Colombo et al., 1999; Oberheim et al., 2006), thus regulating the extracellular K^+^ and Ca^2+^ homeostasis (Hertz and Chen, 2016; Reisin and Colombo, 2002; Verkhratsky et al., 1998). In this view, the classical modular architecture of neurons (Hubel and Wiesel, 1962, 1968), loses its role in segregating the different information, as its activity can be modulated by the distant astrocytic network. Astrocytes in layer I are also responsible for forming the *glia limitans superficialis* and *glia limitans perivascularis*, in charge of protecting the neuronal tissue from cerebrospinal fluid and vascular space (Verkhratsky and Butt, 2023; Webster et al., 2001), a context in which ILAs could have a predominant role. Unlike the horizontal distribution of astrocytes, ILAs appear to be the perpendicular counterpart, responsible for a radial interaction between different layers (Hern’ et al., 2002; Figures 1, 2A–C). This led to the hypothesis that ILAs are specific to primates, facilitating interlayer association to explain the more complex cognitive abilities of this order.

Andriezen was the first to describe “*Caudate Fiber Cells*” as cells typical of the human brain, with the body in the most superficial position and radiating tangential fibers (Andriezen, 1893; Falcone et al., 2019). The following year, Santiago Ramon y Cajal characterized a type of “*cells with long processes*” in the cerebral cortex of a 2-month-old child (Falcone et al., 2019; Ramon y Cajal, 1904). However, no further attention was given to these cells, until 1995, when Colombo et al. (1995), described the presence of GFAP^+^ and Vimentin^–^ cells, in the first layers (I-II) of the frontal cortex of the non-human primates *Cebus apella* and *Saimiri sciureus*. These cells had the typical stellate shape with characteristic processes 300–700 μm long, capable of extending beyond the typical astrocytic single-cell spherical domain to reach lamina IV-V (Colombo, 1996, 2001; Colombo et al., 1995; Falcone et al., 2019). Colombo called these cells *interlaminar astrocytes* (ILAs), distinguishing them from the classical *intralaminar astrocytes* that do not extend beyond their resident layer. The astrocytic nature of ILAs was further confirmed later on—in 2019—when they were shown to express S100β and AQP4, typical astrocytic markers while being negative for neuronal, oligodendrocyte and microglial markers (MAP2, Olig2, and Iba1, respectively) (Falcone et al., 2019). The “re-discovery” of ILAs, supported a change in the perception of the cytoarchitecture of the brain, breaking from the older view that (1) the columnar architecture is specific to neurons (Hubel and Wiesel, 1968) and (2) thin cortex is characterized by astroglia with long processes, while thick cerebral cortex (typical of more complex cognitive abilities) presents small stellate astroglia (Colombo et al., 1997c). Moreover, the absence of ILAs’ typical long processes in the brain of rodents opened new questions on their ontogeny and roles (Colombo et al., 1995).

In 1997, ILAs were described in dissociated cell cultures of fetal monkeys and in the human cerebral cortex of various ages (adults and infants) of both healthy patients and those with neurological disorders. This indicates that ILAs are not a consequence of an inflammatory state of the brain but rather an integral component of it (Colombo et al., 2002; Colombo et al., 1997a; Colombo et al., 1997c), shedding new light on the primate-specific features of these astrocytes. Although the astrocytic organization does not show species-specific morphological features between lissencephalic and gyrencephalic animals, ILAs have a variable morphology between different species (Colombo et al., 2004). In the old-world monkeys (and in particular in humans), ILAs are more numerous, with longer and greater overall complexity compared to the new-world monkeys (Falcone et al., 2019). Interestingly, individual variation in the same species is reduced in the former group compared to the latter (Colombo, 2001).

Even though our understanding of the brain’s structure has improved, we are still far from resolving all the questions related to the brain structure referred to as intellectual abilities.

## Morphology of ILAs

Astrocytes show regional and laminar variation in brain distribution, suggesting their involvement in different local metabolisms or connections (Colombo, 1996). ILAs are characterized by one or more long processes that may enter the deeper cortex, take a diagonal or tangential direction, remain in the superficial laminae, or even not exit their cortical layer (Colombo, 1996; Falcone et al., 2019; Oberheim et al., 2006) (Figures 1, 2A–C). The long processes exiting layer I, where the soma lies, have a tortuous path, creating spiral and twisted configurations (Colombo et al., 1995). Reisin and Colombo (2002) compared different samples of the human cortex from diverse, non-age-matched individuals with different pathological histories, to describe the differences in ILA complexity at regional or individual levels. They identified three main morphologies typical of the long interlaminar processes: (i) mostly rectilinear, following a straight direction, (ii) mostly sinuous, with changes of direction, and (iii) an intermediate morphology, following a single direction with some curvature (Falcone et al., 2019; Hern’ et al., 2002; Reisin and Colombo, 2002; Figures 2C–E).

Interestingly, these long processes do not conform to the classical astrocytic domain, which typically has little to no overlap of processes from different cells. On the contrary, they cross the domains of the protoplasmic astrocytes they encounter, passing in close proximity to their perikaryal (Sosunov et al., 2014). Occasionally, varicosities appear along the interlaminar processes, characterized by a high concentration of GFAP^+^ material (Colombo et al., 1997a). The ends of the longer processes is also varicose-like, creating a club-ending that may increase membrane surface availability (Hern’ et al., 2002; Colombo et al., 1997a; Reisin and Colombo, 2002). Electron microscopic analysis showed the thick GFAP^+^ laminae at the borders of the varicosities and the electron-lucid material, as well as mitochondria in the center (Colombo et al., 1997a). The intensity of GFAP (both in the filaments and in the terminal portions) increases with age in all non-human primates (Colombo et al., 2002). Double labeling of GFAP and Map2 or GFAP and SMI311 (for the IV intermediate filament of the neuronal cytoskeleton) revealed a close relation between the club-ending and neurons (Colombo et al., 1998; Colombo et al., 1997a; Falcone et al., 2019). The complete length of the long ILAs is easily labeled by non-phosphorylated GFAP; using a cocktail of antibodies for GFAP phosphorylated amino acids occasionally covers the total length (Colombo, 2001; Webster et al., 2001).

**FIGURE 3:**
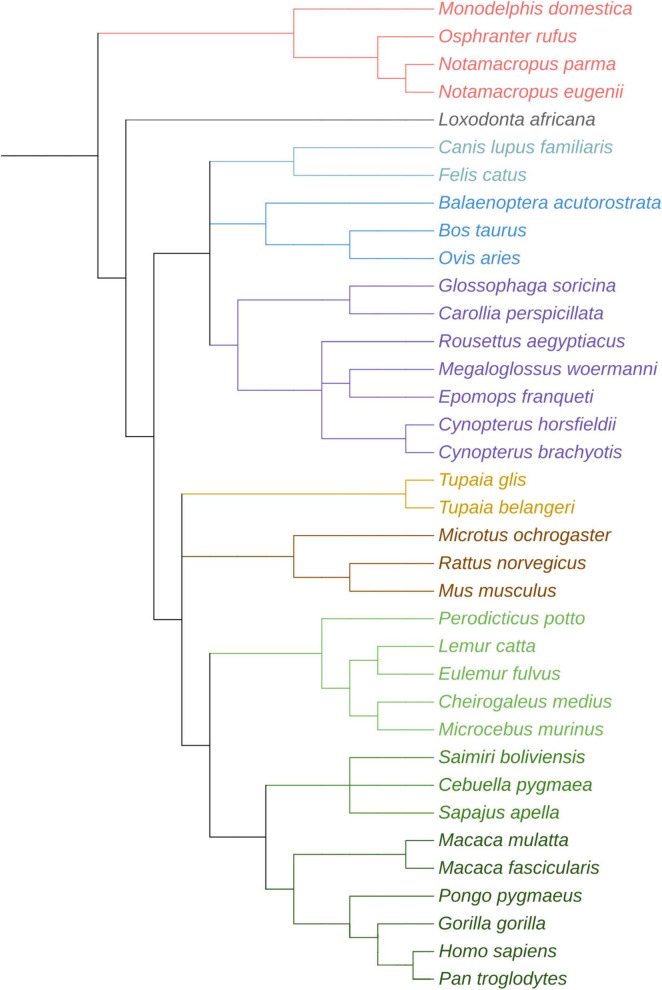
Evolutionary tree showing the phylogenetic relationships of the species mentioned in this review. This tree was created with https://phylot.biobyte.de, species taxonomy codes were obtained from NCBI taxonomy database. Color code indicates closely related mammalian orders or clades. It is as follows: magenta for Marsupials, gray for Proboscidea, light blue for Carnivora, dark blue for Artiodactyls, purple for Chiroptera, yellow for Scadentia, brown for Rodents, green for Primates (specifically, light green for Strepsirrhini, medium green for New and Old World monkeys, dark green for humans and apes).

Histological colorations and electron microscopy observations, suggested that ILAs can be divided into two further subcategories (Colombo et al., 1997a; Falcone et al., 2019): the *pial* ILAs and the *subpial* ILAs. *Pial ILAs* are recognizable by an inverted pyramidal morphology of the soma with the base in direct contact with the *pia mater*. Four to five processes originate from the apex of the pyramid, while a few others are located in the rest of the soma. *Subpial ILAs* differ from the previous ones in their oval-shaped of the perikarya (which remains always in layer I of the cortex, though slightly further from the pia), and contact the *pia mater* via small processes on the upper part of the soma. The perpendicular processes of subpial ILAs may either remain in layer I or extend deeply into the cortex (Falcone et al., 2019). ILAs have been shown to be present in the prefrontal, frontal, temporal, parietal, and occipital human cortex, as well as in the frontal, temporal, occipital cortex of New- and Old-World monkeys (see Table 1 and Figure 3), although with variable distribution (some areas lack long GFAP^+^ processes) and morphology (Colombo et al., 1998; Colombo et al., 1997a; Colombo et al., 2000). Initially, it was believed that *Rodentia*, *Carnivora*, *Ungulata*, *Marsupialia*, *Chiroptera*, and *Insectivora* lacked ILAs, leading to speculations as the progressive evolutionary appearance of the columnar organization of the astrocytes (Figure 3), opposed to the *astroglial syncytium* (Colombo et al., 1998; Colombo, 2001; Colombo et al., 1997c; Colombo et al., 2000), which increases the spatial segregation of incoming signals. However, more recent studies demonstrated that ILAs are present in all mammals, albeit with pronounced morphological differences. Falcone et al. (2019), analyzed brains from Eutheria (*Xenarthra*, *Hyracoidea*, *Proboscidea*, *Rodentia*, *Scandentia*, *Chiroptera*, *Eulipotyphla*, *Carnivora*, *Artiodactyla*), Metatheria (*Diprotodontia* and *Didelphimorphia*) and Primates (*Strepsirrhini*, *Tarsii*, *Platyrrhinii* or New world monkeys, and *Catarrhini* or Old-World Monkeys) (Figure 3). They distinguished ILAs from other astrocytes by the presence of a soma in close proximity with the *pia mater*, and by at least one process traveling perpendicular to the pia. These two characteristics were present in all the mammalian brains analyzed, but the length of the processes varied species by species. For example, ILAs in Marsupialia, Xenarthra, and Rodentia do not exit layer I (and therefore named *rudimentary ILAs*), while in other species, processes reach at least layer II (i.e., *typical ILAs*) (Falcone et al., 2019). Both typical and rudimentary ILAs can have the soma directly in touch with the *pia mater* or through short processes [thus being *pial* or *subpial* ILAs, respectively (Falcone et al., 2019)]. In primates, the long radial process (250–500 μm in non-human primates, up to 1,000 μm in humans) exits layer I and may stop at the next layer (e.g., in *Microcebus murinus*) or cross almost all the cortex, reaching lamina IV and occasionally lamina V (as in humans). Layer VI does not receive fibers from ILAs but instead receives processes directly from the white matter (Colombo et al., 1998, 2000; Colombo et al., 1997a), forming an edge with glial filaments going in opposite directions. *Microcebus murinus*, with its “short” processes, shows an intermediate astroglia phenotype between rudimentary ILAs and typical ILAs, with GFAP^+^ long processes that are limited in length (250–300 μm), rarely exit layer II, and are localized in circumscribed areas (striatal, parietal, and orbitofrontal cortices) (Colombo et al., 1998, 2000; Colombo et al., 1997a). No correlation was found between the thickness of the cortical layer I (which is higher in *Microcebus* than in other primates, see Colombo et al., 1998) and the length of ILAs processes (Colombo, 2001; Colombo et al., 1998). The diameter of ILA processes increases with age, particularly at the level of the club-endings that are more GFAP^+^ positive in older individuals, as shown in the brains of *Macaca mulatta* and *Papio hamadryas* at different ages (Colombo et al., 1998). Moreover, there is a slight difference in ILA density and complexity between species. For an objective measurement of the complexity of cells belonging to different species, it is more and more used the complexity index (CI), which takes into consideration the number of primary processes, the number of total processes, and their lengths [calculated as (Σ terminal orders + number of terminals) × (total process length/number of primary branches), where the number of “terminal orders” is the number of branches met going backward from the terminal to the soma (SheikhBahaei et al., 2018). Humans and non-human primates have the highest density, and complexity index], as opposed to marsupials, which show the lowest density and complexity of ILAs (Falcone et al., 2019). The difference in density between humans and Marsupialia is two-fold, and the complexity index difference is 17-fold.

**TABLE 1:** Summary of the distribution and morphological hallmarks of interlaminar astrocytes (ILAs) and varicose-projection astrocytes (VP-As) across species.

Species	Common name	Rudimentary and/or typical ILAs	ILA/VP-A morphology and location	References
**Primates**				
**Strepsirrhini**				
*Cheirogaleus medius*	Lesser dwarf lemur	Typical pial ILAs and rudimentary subpial ILAs	Both pial and subpial ILAs are present in all the cortex	Falcone et al., 2019
*Lemur catta*	Ring-tailed lemur	Typical pial ILAs and rudimentary subpial ILAs	Both pial and subpial ILAs are present in all the cortex	Falcone et al., 2019
*Microcebus murinus*	Gray mouse lemur	Typical ILAs	Processes rarely cross layer II and can be straight or tortuous; they show an intermediate astroglia phenotype between rudimentary ILAs and typical ILAs	Colombo et al., 1998
*Perodicticus potto*	Potto	Typical pial ILAs and rudimentary subpial ILAs	Both pial and subpial ILAs are present in all the cortex	Falcone et al., 2019
**New world monkeys**				
*Eulemur fulvus*	Brown lemur	Rudimentary ILAs	Glial structure looks similar to the rodents’ one	Colombo et al., 2000
*Saimiri boliviensis*	Bolivian squirrel monkey	Typical ILAs that reach lamina III	Reduced in number and length compared to humans. Between the New World Monkeys, Saimiri, shows a lamina I with the most consistent mesh of GFAP^+^ astrocytes, with long processes arriving also to 300 um and a honeycomb-like distribution	Colombo et al., 1999, Colombo et al., 2000, Oberheim et al., 2009
*Sapajus apella*	Tufted capuchin	Typical ILAs that reach lamina III	ILAs are localized in patches in the striatum with a honeycomb-like distribution. The first layer shows a mesh of GFAP^+^ astrocytes with the longest processes between the New World Monkeys. Surgical visual deprivation showed a decay of ILAs	Colombo et al., 1999, Colombo et al., 2000, Colombo et al., 2001, Reisin and Colombo, 2002
**Old world monkeys**				
*Macaca mulatta*	Rhesus macaque	Typical pial and subpial ILAs, rudimentary subpial ILAs	ILAs are reduced in number and length compared to humans. The processes are parallel and straight but get tortuous toward the terminal portions. The distribution of typical and rudimentary ILAs are similar in the *Macaca fascicularis*	Oberheim et al., 2009, Falcone et al., 2019
*Macaca fascicularis*	Crab-eating macaque	Typical pial and subpial ILAs, rudimentary subpial ILAs	In the dorsal cortex there are both the typical pial and rudimentary subpial ILAs, while in the ventral cortex, there are just typical ILAs (both pial and subpial). Typical ILAs can reach layers II and III, and sometimes even layer IV.	Falcone et al., 2019
**Species**	**Common name**	**Rudimentary and/or typical ILAs**	**ILA morphology and location**	**References**
**Great apes and human**				
*Gorilla gorilla*	Western gorilla	Typical ILAs	ILAs are well defined in palisade and spread in all the cortex; VP-As present in deeper layers and WM.	Colombo et al., 2004, Falcone et al., 2019
*Homo sapiens*	Human	Typical ILAs until layer IV	Humans present ILAs with the longest processes (even 1 mm) that reach lamina IV. They increase in GFAP immunoreactivity with age. VP-As present in deeper layers and WM.	Colombo et al., 2001, Webster et al., 2001, Colombo et al., 2002, Falcone et al., 2019
*Pan troglodytes*	Chimpanzee	Typical ILAs	ILAs show long processes able to exit layer I, not as long as in humans. Not all samples analyzed showed the presence of the typical interlaminar palisade. This condition was always associated with evidence of astrogliosis. This species presents just typical ILAs in all the cortex. VP-As present in deeper layers and WM.	Colombo et al., 2004, Oberheim et al., 2009, Falcone et al., 2019
*Pongo pygmaeus*	Bornean orangutan	Typical ILAs	They show a well-defined typical intralaminar palisade, with just typical ILAs in all the cortex. VP-As present in deeper layers and WM.	Colombo et al., 2004, Falcone et al., 2019
**Proboscidea**				
*Loxodonta africana*	African savanna elephant	Typical pial ILAs with the co-presence of rudimentary subpial ILAs in the ventral cortex	Typical ILAs are present both in dorsal and ventral area, while the rudimentary subpial ILAs are present just in the ventral cortex	Falcone et al., 2019
**Rodentia**				
*Cynopterus horsfieldii*	Horsfield’s fruit bat	Typical ILAs that reach layers II and III		Colombo et al., 2000
*Cynopterus brachyotis*	Lesser short-nosed fruit bat	Typical ILAs that reach layers II and III		Colombo et al., 2000
*Microtus ochrogaster*	Prairie vole	Rudimentary ILAs–just pial	Present just in the dorsal cortex	Falcone et al., 2019
*Mus musculus*	Mouse	Rudimentary ILAs–both pial and subpial	Present both in the dorsal and ventral cortex	Colombo et al., 1995, Oberheim et al., 2009, Falcone et al., 2019
*Rattus Norvegicus*	Norwegian Rat	Rudimentary ILAs–both pial and subpial	Present both in the dorsal and ventral cortex	Colombo et al., 2001, Falcone et al., 2019
**Chiroptera**				
*Carollia perspicillata*	Seba’s short-tailed bat	Prevalently rudimentary ILAs, with presence of typical ILAs in the ventral cortex	Pial ILAs are both dorsal and ventral, while subpial ILAs are just ventral	Falcone et al., 2019
*Glossophaga soricina*	Pallas’s long-tongued bat	Prevalently rudimentary ILAs, with presence of typical ILAs in the ventral cortex	Pial ILAs are both dorsal and ventral, while subpial ILAs are just ventral	Falcone et al., 2019
*Epomops franqueti*	Franquet’s epauletted bat	Prevalently rudimentary ILAs, with presence of typical ILAs in the ventral cortex	Pial ILAs are both dorsal and ventral, while subpial ILAs are just ventral	Falcone et al., 2019
*Megaloglossus woermanni*	Woermann’s bat	Prevalently rudimentary ILAs, with presence of typical ILAs in the ventral cortex	The only Chiroptera analyzed that do not show any presence of subpial ILAs	Falcone et al., 2019
*Rousettus aegyptiacus*	Egyptian rousette	Prevalently rudimentary ILAs, with presence of typical ILAs in the ventral cortex	Pial ILAs are both dorsal and ventral, while subpial ILAs are just ventral	Falcone et al., 2019
**Carnivora**				
*Canis familiaris*	Dog	Rudimentary ILAs—occasionally entering layer II		Colombo et al., 2000
*Felis catus*	Domestic cat	Prevalently rudimentary ILAs, with typical pial ILAs in the ventral cortex	Rudimentary ILAs are present in both the dorsal and the ventral cortex, with typical pial ILAs just in the ventral cortex	Falcone et al., 2019
**Artiodactyla**				
*Balaenoptera acutorostrata*	Minke whale	Just pial ILAs, both typical and rudimentary	Present both in the dorsal and ventral cortex	Falcone et al., 2019
*Bos taurus*	Cattle	Rudimentary ILAs		
*Ovis aries*	Sheep	Rudimentary ILAs	There are just rudimentary pial ILAs in the dorsal cortex. No other type of ILAs were detected	Falcone et al., 2019
**Marsupialia**				
*Monodelphis domestica*	Gray short-tailed opossum	Rudimentary pial ILAs	Present just in the ventral area of the cortex	Falcone et al., 2019
*Notamacropus eugenii*	Tammar wallaby	Rudimentary pial ILAs		
*Notamacropus parma*	Parma wallaby	Rudimentary pial ILAs	Present just in the ventral area of the brain	Falcone et al., 2019
*Osphranter rufus*	Red kangaroo	Rudimentary pial ILAs	Present just in the ventral area of the brain	Falcone et al., 2019
**Scandentia**				
*Tupaia belangeri*	Northern tree shrew	Rudimentary pial and subpial ILAs, with the co-presence of typical ventral ILAs	The rudimentary ILAs are spread in all the brain, while the typical pial ILAs are present just in the ventral cortex	Falcone et al., 2019
*Tupaia glis*	Common tree shrew	Rudimentary ILAs–long processes that do not exit layer I	The first layer is thick. ILAs do show long processes, but they are not long enough to reach layer II	Colombo et al., 2000

ILAs exhibit a patch distribution in the brain: GFAP immunolabeling of the medial portion of the striatal cortex of *Cebus apella* and *Saimiri boliviensis* monkeys show alternating zones of density with long processes extending from layer II. In horizontal sections, the same labeling displays a honeycomb-like distribution, with the diameter of the surrounding area similar to the pyramidal dendritic bundles located in layer II and III, suggesting that ILA may be involved in the spatial segregation of neural modules (Colombo et al., 1999). From this perspective, ILAs form a perpendicular network that contrasts with the horizontal one created by canonical astrocytes. This patchy distribution of ILAs, in concomitance with neural columns, may correlate to neuronal distribution in an attempt to preserve the division of information in the brain (Colombo et al., 1999).

## Development and role of ILAs

The origin and roles of ILAs remain some of the most intriguing and challenging questions in the glial biology field. Morphological description using immunological techniques gives crucial information on the timing of appearance and morphological changes during prenatal and postnatal development, but only allows for speculative hypotheses regarding the origin and functions, which still constitute an exciting unresolved mystery. Here, we provide an overview of the debates that are still ongoing.

The initial hypothesis regarding the origin of ILAs was proposed by Colombo (2001), Colombo et al. (1997a,c), considering another type of non-neuronal cells characterized by long processes: the radial glial cells (RG), present in early developmental stages of the brain, even in less complex vertebrates. Currently, there are four main hypotheses on the embryonic development of ILAs (Falcone et al., 2021):

1. ILAs express markers typical of the inner RG (Cryab) and outer RG (Hopx), as well as a set of proteins that are expressed by the RG during early development (Pax6, Sox2, and Nestin), suggesting a possible direct transformation of these cells into immature ILAs;
2. ILAs could derive from RG indirectly, passing through an intermediate progenitor cell that still expresses stem cell markers as Pax6 and Sox2;
3. The intermediate progenitor can also evolve into cortical astrocytes, that eventually differentiate into ILAs, leading to a four-stage maturation; Indeed, ILAs increase in number postnatally, which could derive from cortical astrocytes expressing the proliferative marker Ki67.
4. ILAs originate from other regions (such as the ventral forebrain) and migrate to their final locations. Even if there is no evidence yet, we cannot rule out this hypothesis.

These hypotheses are not mutually exclusive, as there is evidence supporting each of them. However, the fact that the *typical* ILAs are not present in all mammals suggests they might be a distinct cell type compared to the *rudimentary* ones (Colombo et al., 1995) or that they are the only ones derived from RG as a gain of function in mammals with more complex cognitive abilities. Indeed, mouse *rudimentary* ILAs only partially overlap the expression of the set of markers of *typical* ILAs and do not increase in morphological complexity during life as *typical* ILA do (Falcone et al., 2019). Nevertheless, they seem to derive from a common ancestor, as immature typical ILAs strongly resemble mature rudimentary ILAs (Falcone et al., 2019).

Clues on the divergent ontology of ILA from RG are:

1. The cell somata is localized in layer I, in contrast to the position of RG somata (Colombo et al., 1997a).

2. Several short processes are typical of differentiated astrocytes (Colombo et al., 1997a).

3. ILAs maturate postnatally, whereas RG transforms into astrocytes in the late pre-natal phase (Colombo et al., 1997a), suggesting different existence windows for RG and ILAs.

There is still no certainty about ILAs development in humans. A first study published in Colombo et al. (2002), timed the development of ILAs to the postnatal period, much later than *intralaminar* astrocytes. They did not find the classical ILA processes in the cortex 16 days after birth but described remnants of RG cells (Colombo et al., 2005). According to this study, ILAs start to be visible between 20 and 40 days after birth; at 40 days, the perpendicular long GFAP-positive processes are visible in most cortical areas, although they typically do not exit layer I. Growth in length is completed six months after birth (Colombo et al., 2005; Colombo et al., 1997c). Colombo and his group confirmed their presence also in a 7-year-old child’s brain specimen, with peculiar Vimentin positivity suggesting ongoing maturation, and in adults aged 14, 30, 39, and 84 years (Colombo et al., 1997a) demonstrating that ILAs are not regressive (Colombo et al., 1997c).

In contrast, Sosunov et al. (2014), found pial-based, GFAP^+^/CD44^+^ astrocytes at 26 weeks of gestation and confirmed their presence also in young brains of 2-year-old children, agreeing with Colombo that ILAs belong to the normal astrocytic developmental program. Falcone et al. (2021), showed the presence of newborn ILA somata (positive to GFAP and S100β) in contact with the *pia mater* during early prenatal stages in rhesus macaque and humans. These early ILAs can locally proliferate (as indicated by positivity for Ki67, a mitotic marker) until the late second trimester and increase in complexity during the postnatal period, strongly supporting the argument that ILAs belong to the embryonic development.

An interesting experiment performed by Colombo et al. (1997b), suggests a role for ILAs in responding to insults. Following the intracranial injection of KCl (5 and 50 mM) in the striatal cortex leads, a short-term rearrangement of the long processes of ILAs was observed near the injection site. This included increased GFAP immunoreactivity, enhanced expression of Vimentin at the club-ending, and the presence of numerous thickenings. Conversely, long-term effects of the mechanical lesion showed a replacement of ILAs with reactive astrocytes, creating a sort of scar along the lesion borders (Colombo et al., 1997b). The columnar morphology of ILAs may indicate a role in trafficking and integrating information from the superficial layers to the deep ones, as well as in horizontal interactions via short processes that do not exit layers I and II (Colombo, 1996). This vertical arrangement of ILAs intrigued Colombo and Reisin (Colombo and Reisin, 2004) who speculated about a possible role as a horizontal barrier in the diffusion of molecules. One hypothesis suggests a role in potassium regulation on a broader scale compared to the classical astrocytic network. This theory is supported by the sensitivity of ILAs to local KCl application, which leads to the appearance of varicosities along the interlaminar process (Colombo et al., 1997b). The formation of fascicules in a honeycomb pattern, visualized in tangential sections of the human brain (Colombo et al., 1998), suggests a role in the “columnization” of Ca^2+^ waves around granular neurons, leading to *cylindrical-like* waves that follow the morphology of ILAs long processes (Colombo and Reisin, 2004; Hern’ et al., 2002). The involvement of ILAs in response to insults is further confirmed by their degeneration in neurodegenerative disorders such as Alzheimer’s disease (see next chapter).

An elegant study on the possible roles of ILAs was conducted by Rao et al. (2016). They compared the microRNA (miRNA) expression profile of astrocytes directly extracted from human brains without known pathologies using laser capture microdissection (with GFAP and astrocytic morphology as discriminant markers). miRNA are small non-coding RNAs responsible for the post-transcriptional regulation of gene expression, providing insight into differentially expressed genes. ILAs were found to have a unique miRNA profile compared to the protoplasmic and fibrous astrocytes. There was a low or undetectable expression of miRNA-regulating genes linked to cell proliferation, development, migration, angiogenesis, and pro-inflammatory processes, suggesting high stability of these cells, which do not appear deeply involved in metabolic homeostasis. For instance, the low expression of miR-34a (which induces astrogliogenesis) is probably linked to the stability of ILA; while the low expression of miR-449 (a regulator of microtubule dynamics in cell proliferation) is linked to their typical architecture. On the contrary, there was an abundance of anti-inflammatory miRNA (as miR-146, which coincides with the limited expression of pro-inflammatory miR-338 and miR-365) and modulators of GFAP expression (as miR-125b), supporting the hypothesis of ILA as a “first-line defense” against external insults (Rao et al., 2016). This analysis revealed that ILAs have an expression profile distinct from other astrocytes, indicating more specific and restrictive roles.

Other two potential physiological roles of ILAs could be in the regulation of synapse formation and functions and in the glymphatic system. First, although no functional experiments have been done on ILA involvement in synaptogenesis, ILA processes have been shown to contact MAP2+ neuronal processes (Falcone et al., 2019) in postmortem rhesus macaque brains, and VGLUT1+ synapses in a chimeric model with human astrocytes transplanted into mice (Padmashri et al., 2021). Taken together, these observations point to the role of ILAs in regulating synaptic formation and functions. Second, ILA membranes have been shown to express high levels of Aqp4 marker, also around the cell body which is in strict contact with the pia surface (Falcone et al., 2019). This strongly suggests that ILAs are involved in the glymphatic system and the regulation of water homeostasis at the blood-brain barrier and at the pia interface in particular. Interestingly, as a support of a potential involvement of ILAs in blood-brain barrier regulation, it has been shown that ILA processes do also directly contact blood vessels in the prefrontal cortex of rhesus macaque brains (Falcone et al., 2019).

Finally, little is known about the molecular identity of ILAs. A recent preprint showed that they specifically express higher levels of Myoc gene compared to astrocytes residing in other cortical layers, in both mice and humans (Hasel et al., 2023). Further transcriptomics studies will be needed to identify ILA specific molecular signatures.

## ILAs in brain diseases

Glia is involved in many brain disorders, ranging from psychiatric to neurodegenerative (Endo et al., 2022; Aamodt, 2007), with controversial roles: they contribute to neurodegeneration, inflammation, and formation of glial barrier (Amadio et al., 2010) as well as maintaining homeostasis and restoring damaged tissues (Amadio et al., 2010; Endo et al., 2022). Astrocytes can activate a series of answers in the presence of pathologies, forming reactive astrocytes, atrophy due to loss of function, degenerating and dying, or developing aberrant astrocytes (astrocytopathies) (Verkhratsky et al., 2023). Here, we summarize the involvement of ILAs in different brain diseases as an approach to understanding their role in a healthy brain.

Major mental illnesses such as schizophrenia, bipolar disorders, and major depression affect behavior, are led to the misconception that they are solely due to abnormal neuronal activity. Recent studies have shown that these disorders are also connected to significant abnormalities in glial functions and reduced glial density in several brain areas (Webster et al., 2001). However, Webster et al. (2001), found a significant specific reduction in the four isoforms of GFAP, specifically in the perivascular glia, suggesting ILAs are not directly involved. On the other hand, another study by Amin et al. (2018) on depression, suggests a direct involvement of ILAs in patients with depressive symptoms. Depression, the most prevalent psychiatric disorder, is difficult to evaluate and treat due to the lack of identified responsible genes. The difficulty of treatment is also associated with a lack of identification of genes responsible for it in several whole genome studies performed over the years. Amin et al. (2018) focused on the genetically isolated Erasmus Rucphen Family (ERF) community, identifying a rare mutation in the RCL1 gene (an RNA 3′-terminal phosphate cyclase-like protein). Homozygous carriers of the mutated form of RCL1 in the ERF population showed high scores of depressive symptoms and a diagnosis of Major Depressive Disorders, compared to heterozygous patients with milder symptoms. In postmortem brains, RCL1 is expressed specifically in the nuclear region of neurons and in the long processes of ILAs (and in no other protoplasmic astrocytes), leading to the hypothesis of an active role of ILAs in depression. However, further studies are needed to validate this hypothesis (Amin et al., 2018).

While *intralaminar* astrocytes react to insults, with varying levels of astrogliosis (i.e., upregulation of GFAP as mRNA and protein, soma hypertrophy, increased number of processes, and loss of the organization in domains) (Escartin et al., 2021), ILAs are susceptible (Edler et al., 2023; Oberheim et al., 2006), but not directly involved in the reaction against the disorder’s development (Colombo, 2001; Colombo et al., 2002). Patients with Alzheimer’s disease (AD) show a reduction in glial density at the blood-brain barrier (Webster et al., 2001), and an increase in astrogliosis and astrocyte recruitment at the inflammation sites (Edler et al., 2023). ILAs lose their palisade structure, although they do not directly co-localize typical neuritic plaques (Colombo et al., 2002; Edler et al., 2023). Evidence of Ca^2+^ signaling disruption in AD’s early stages has been observed in both neurons and astrocytes, even before the appearance of the typical cognitive decline (Garwood et al., 2013). An aberrant increase in *Calpain-10* protein (a protease involved in Ca^2+^ homeostasis) and a reduction of CamKII (a Ca^2+^ signaling kinase) correlates with disease progression, particularly in ILAs (Garwood et al., 2013). Studies on AD in primates (marmosets, macaques, gorillas, and chimpanzees) showed species-specific astrocyte and microglia reactions during different astrogliosis stages, due to the different stages of the disorder (Edler et al., 2023). In chimpanzees, disruptions of the interlaminar palisade resemble those in human patients, associated with Aβ plaques or high levels of tau phosphorylation, often without microglia activation.

Multiple sclerosis (MS) starts as an autoimmune inflammatory reaction against myelin, leading to oligodendrocyte and neuronal axon degeneration in both white and gray matter (Amadio et al., 2010). Astrocytes play key roles in both the destructive and regenerative phases of MS, mostly thanks to the purinergic signaling. Amadio et al. (2010) localized the purinergic receptor P2Y12 to myelin and ILAs but not protoplasmic astrocytes, suggesting a role in long-distance signaling within the different cortical layers. This signaling is disrupted by the degeneration of the interlaminar processes in MS patients (Amadio et al., 2010).

Among neurodegenerative disorders, human prion diseases are a group of progressive and fatal conditions caused by pathological aggregates of misfolded prion protein which lead to protein deposits, neuronal death, vacuolation, and gliosis (Garcés et al., 2021). Astrocytes are the first cells to exhibit aggregates of the scrapie form of the prion protein, resulting in profound alterations in astrocytic morphology. In particular, ILAs show disorganized palisades and varicosities along their processes and terminal buttons, with features similar to those observed in AD brains (Garcés et al., 2021).

The pathogenesis of neurodegenerative disorders may also be linked to exposure to trace metals (Li et al., 2021, 2024). Mercury (Hg) exposure, for instance, has been associated with the development of AD, MS, and Amyotrophic Lateral Sclerosis (ALS). The role of astrocytes in the spreading of Hg into the brain has been studied on a young man of 24 years old who injected himself intravenously with metallic mercury (Pamphlett and Kum Jew, 2018; Pamphlett and Waley, 1996). *Post-mortem* immunostaining of sections of his brain showed that Hg was distributed mainly in all five subclasses of astrocytes: in the perikarya of the glia limitans, the long processes of ILAs, the perikarya of protoplasmic and fibrous astrocytes, and the varicosities of the VP-As. The likely mechanism of Hg spread is from the perivascular astrocytes and those in contact with the subarachnoid cerebrospinal fluid to other brain cells via gap junctions, extracellular vesicles, or other intercellular connections. Notably, only oligodendrocytes in the gray matter, and not those in the white matter, were found with Hg deposits. Another possible mechanism involves Hg movement from neurons to astrocytes, as an attempt to sequester Hg and mitigate its neurotoxic effects. In either case, astrocytes are the primary targets of this heavy metal, contributing to a toxic condition that exacerbates the development of severely impacting neurodegenerative disorders (Pamphlett and Kum Jew, 2018). Astrocytes have also been found to be active reservoirs for trace metals such as iron, copper, manganese and zinc, in order to fulfill nervous system demand of such metals and thus regulating CNS iconostasis (Li et al., 2024).

Down’s syndrome (DS) is another pathology in which ILAs have been analyzed, encompassing cases from 3 months old to 69 years old (Colombo et al., 2005; Colombo and Reisin, 2004). While no differences are observed between DS and healthy patients at birth, by 6 months of age, there is a general alteration of the tortuosity and parallelism typical of ILA long processes. These changes become particularly visible by the end of the first year of life (Colombo et al., 2005; Colombo and Reisin, 2004). In adults, the destruction of the typical ILA palisade is even more pronounced, especially in the presence of amyloid plaques, with variability reflecting the individual severity and clinical progression of the syndrome. DS often co-occurs with AD’s type of dementia, and in such cases, the ILA palisade is localized in “patches,” mirroring the progression of AD patients without DS. As dementia advances, the ILA palisade is almost completely destroyed, accompanied by an increase in astrogliosis in the astrocytic network (Colombo et al., 2005).

Another syndrome connected with ILA is epilepsy, a neurological condition affecting millions of people worldwide and often refractory to treatment. Recent studies have shown that regions with high spiking activity are linked with decreased GFAP expression in certain brain areas, high levels of astrogliosis (Chaslin’s gliosis), and loss of the typical astrocytic domain (Zhou et al., 2023). Interestingly, patients with epileptic attacks have a higher number of ILAs in the first layer of the temporal lobe, unlike other neurodegenerative disorders such as AD, and the long processes in these patients do not appear damaged, especially in those with treatable epilepsy.

For a comprehensive understanding of the role of astrocytes in pathologies, it is important to mention the astrocytes-derived extracellular vesicles (AEVs). AEVs are released in high numbers in resting conditions as horizontal messengers, completing the abilities of astrocytes. However, in pathologies or inflammatory insults, AEVs show a double face. Indeed, they can exert a neuroprotective role [by moving neurotoxic molecules out of the cell (Falker et al., 2016)] together with a neurotoxic effect [by helping the spread of disease-associated molecules such as amyloids, and/or prions (Falker et al., 2016; Zhao et al., 2021)]. For instance, in AD and PD, astrocytes try to balance the excessive production of amyloids, by increasing the release of AEVs containing molecules that are supposed to inhibit the neurotoxic effects of the plaques, but also induce apoptosis on neurons (Leggio et al., 2021; Marchetti et al., 2020). In ALS, mutated AEVs are responsible for the neuronal death. While it is clear that AEVs can be useful markers for the diagnosis of neurological disorders, most of their consequences in pathologies are still unknown (Zhao et al., 2021). Taken together, these data suggest a sensitivity of ILAs to stressful conditions (Hern’ et al., 2002).

## Varicose-projection astrocytes

Varicose-projection astrocytes (VP-As) were first described by Oberheim et al. (2009). VP-As are a distinct class of astrocytes positive for GFAP, with their soma localized in the deep layers V and VI, and white matter. These cells are characterized by the presence of short processes around the soma and one to five long processes with varicosities spaced approximately every 10 μm along the length of the fibers.

Similarly to ILAs, VP-As do not respect the single astrocytic domain rule of neighboring astrocytes and extend processes toward long distances, however without crossing the layer boundaries (Oberheim et al., 2009; Pereira and Furlan, 2010). Initially referred to as “human-specific” astrocytes upon their first discovery, it was later found by Falcone et al. (2022) that VP-As are also present in apes (chimpanzee, bonobo, orangutan, gorilla, gibbon, and siamang), but not in other primates. Intriguingly, VP-As were found to be “individual-specific,” meaning they were not present in all the individuals within the same species, but only in some of them, enforcing the hypothesis of their origin as linked to specific brain conditions (Falcone et al., 2022; Pereira and Furlan, 2010; Sosunov et al., 2014). VP-As seem to be linked to ILAs: they express similar markers (GFAP, CD44, Aqp4, Aldh1L1, and S100β). Additionally, VP-A’s presence co-occurre together with the appearance of varicosities along ILA perpendicular fibers, strengthening the hypothesis that VP-As may arise from a morphological change in resident astrocytes in response to specific insults (Falcone et al., 2022). These varicosities may be caused by age (no VP-As were found in fetal brains or patients of 5 or 10 years old), stress, traumatic injuries (that are often connected with the presence of varicosities on ILAs), or acute inflammatory events (even though it was not found activated microglia in the samples studied by Falcone et al. (2022)). No species-related differences in morphology were noticed between VP-As belonging to humans and great-apes and they are localized in all the areas of the brain (Falcone et al., 2022). Many more studies are required to understand the exact role of these astrocytes in apes and humans, in health and in disease.

## Models and methods

Immunocytochemistry (IHC) first and immunofluorescence (IF) later, are the classical methods used for detailed morphology, contact detection, and target characterization. These techniques need to be supplemented with other approaches to gain more insights into ILAs functions. The spatial configuration of ILAs can give clues on their local or regional interactions (Reisin and Colombo, 2002). Fractal dimension (FD) and compression analysis (CA) are used, respectively, to describe the cellular complexity and coverage of the regional space. FD was a useful tool for a better comprehension of the reaction of astrocytes to pathologies. Indeed, FD applied on astrocytes in the presence of pathologies causing dementia such as Alzheimer’s disease, Ischemia, or Hemorrhage demonstrated morphological changes (Pirici et al., 2009): astrocytes react differently to different insults. They surround the core of the lesion in hemorrhage and spread the reaction diffusely in ischemia. In contrast, they surround the neuritic dense plaques in Alzheimer’s disease (Pirici et al., 2009). Therefore, fractal dimension analysis could be a valuable tool to have clues on the consequences on ILAs in the presence of pathologies. The first attempt was performed by Reisin and Colombo (2002), trying to describe three different morphologies for the long interlaminar processes, suggesting regional adaptation of ILAs (Reisin and Colombo, 2002). However, while FD was not adequate to describe the complexity of the interlaminar processes, CA (taken a region of interest, it is measured by the variable *k* = absolute length of the process / effective length of the process) appeared to be an effective method to analyze the different tortuosity of ILA’s processes.

DiOlistic labeling is a remarkably effective method for fluorescent, cell membrane labeling. Its major advantage over classical IF is that it labels all astrocytic processes, including the most distal ones. Using two spectrally distinct lipophilic dyes for two adjacent cells allows visualization of borders and, therefore, astrocytic tiling and domain organization (Oberheim et al., 2009). Lipophilic dye tracing is also very useful for sparse labeling and single-cell morphology reconstructions.

Other interesting techniques used for studying ILAs include:

1. “Tissue printing” method for the dish culture of ILA, where ILAs are detached from fresh slabs of the brain onto specific substrates and maintained stable for at least 24 h (Colombo et al., 2001).
2. Engraftment of human-specific ILAs in the first layer of the mouse cortex (Padmashri et al., 2021), also known as the creation of chimeric mice. This innovative technique allows the study of cells that retain the intrinsic properties of human ILAs (such as process length and tortuosity, the presence of club-ending and typical interlaminar markers) and integrate into the host environment, contacting synapses, capillaries, and the pia.
3. Study of the morphological features of ILAs (as well as other astrocytes) through the software for three-dimensional reconstructions, such as Neurolucida and Imaris, which can give information about the spatial domain and territorial volume covered by each cell, morphological complexity, and contacts with other cortical structures (Zhou et al., 2023).

The advent of the -omics (as the spatial transcriptomics and the single cell nuclei) and the employment of primate organoids and chimeric mice will likely open new intriguing avenues to identify the molecular signatures of such astrocytes and their functions in the primate brain.

## Conclusion

In conclusion, this review highlights the significant roles and diverse characteristics of a type of astrocytes with primate-specific features: the ILAs, and a special mention to VP-As. The involvement of ILAs and VP-As in numerous neurological and neurodegenerative disorders, such as AD, DS, epilepsy MS, and human prion diseases, suggest a key role for them in both the maintenance of normal brain function and the pathogenesis of these conditions. ILAs and VP-As are not only sensitive to various brain insults but also possibly adapt their morphology and function in response to specific diseased conditions. Future research focusing on these astrocytic subtypes will be crucial for unraveling their exact contributions to brain health and disease, potentially opening new avenues for therapeutic strategies. Last but not least, investigating primate-specific functions of such astrocytes may help us better understand the evolution of the primate brain and primate-associated complex cognitive abilities.
